# DFA-UNet: dual-stream feature-fusion attention U-Net for lymph node segmentation in lung cancer diagnosis

**DOI:** 10.3389/fnins.2024.1448294

**Published:** 2024-07-15

**Authors:** Qi Zhou, Yingwen Zhou, Nailong Hou, Yaxuan Zhang, Guanyu Zhu, Liang Li

**Affiliations:** ^1^Department of Radiotherapy, The Affiliated Hospital of Xuzhou Medical University, Xuzhou, China; ^2^School of Medical Imaging, Xuzhou Medical University, Xuzhou, China

**Keywords:** ultrasound elastography, mediastinal lymph nodes, semantic segmentation, attention mechanism, deep learning

## Abstract

In bronchial ultrasound elastography, accurately segmenting mediastinal lymph nodes is of great significance for diagnosing whether lung cancer has metastasized. However, due to the ill-defined margin of ultrasound images and the complexity of lymph node structure, accurate segmentation of fine contours is still challenging. Therefore, we propose a dual-stream feature-fusion attention U-Net (DFA-UNet). Firstly, a dual-stream encoder (DSE) is designed by combining ConvNext with a lightweight vision transformer (ViT) to extract the local information and global information of images; Secondly, we propose a hybrid attention module (HAM) at the bottleneck, which incorporates spatial and channel attention to optimize the features transmission process by optimizing high-dimensional features at the bottom of the network. Finally, the feature-enhanced residual decoder (FRD) is developed to improve the fusion of features obtained from the encoder and decoder, ensuring a more comprehensive integration. Extensive experiments on the ultrasound elasticity image dataset show the superiority of our DFA-UNet over 9 state-of-the-art image segmentation models. Additionally, visual analysis, ablation studies, and generalization assessments highlight the significant enhancement effects of DFA-UNet. Comprehensive experiments confirm the excellent segmentation effectiveness of the DFA-UNet combined attention mechanism for ultrasound images, underscoring its important significance for future research on medical images.

## 1 Introduction

Lung cancer is one of the malignant tumors with the highest morbidity and mortality rates worldwide (Detterbeck et al., 2016; Siegel et al., 2023). The choice of treatment is closely related to cancer staging, determining whether the lymph nodes are involved is one of the key factors in clarifying the cancer staging (Asamura et al., 2015; Taylor et al., 2023). Numerous studies (Gu et al., 2017; Wang et al., 2018; Zhang et al., 2019; Wang B. et al., 2021; Wang R. et al., 2021) have demonstrated that compared with traditional ultrasound imaging, bronchial ultrasound elastography (BUE) can provide more accurate information on mediastinal lymph nodes, reflecting the hardness information of lymph node tissues with different colors, which has a higher diagnostic value (Oglat and Abukhalil, 2024).

Ultrasound elastography (UE) is a novel ultrasound diagnostic technology that has rapidly developed in recent years. It utilizes dynamic imaging to measure tissue hardness (Zhang et al., 2019; Cui et al., 2022), allowing for non-invasive diagnosis of diseased tissues by analyzing the differences in hardness between various tissues. Currently, most UE used in endoscopy employs strain force elastography. This technique operates on the principle that softer and harder tissues deform differently under the same external force (Sigrist et al., 2017). Generally, tissues with lower elasticity coefficients exhibit greater displacement and deformation, appearing green; tissues with higher elasticity coefficients exhibit less displacement, appearing blue; and tissues with intermediate hardness appear reddish-blue or reddish-green. Since malignant lymph nodes are harder than benign ones, assessing the hardness of a lesion by measuring the proportion of the blue area within it can help identify benign and malignant lesions (Sun et al., 2017). Therefore, accurate localization and segmentation of mediastinal lymph nodes based on BUE images are crucial steps in lung cancer diagnosis and treatment (Wang B. et al., 2021; Wang R. et al., 2021).

Currently, professional doctors are typically required to manually segment lymph nodes in BUE images. This process is not only time-consuming and labor-intensive but also subject to inter-individual differences among doctors, leading to subjective biases and potential omission of important features. Consequently, the same image can result in varying analyses and evaluations, causing segmentation errors. Therefore, developing automatic segmentation methods for lymph nodes in UE images is of great significance (Li and Xia, 2020; Tan et al., 2023).

With the continuous development of computer vision technology, the application of semantic segmentation in medical images has become increasingly important. Combining artificial intelligence with medical imaging to enable intelligent-assisted diagnosis has become an inevitable trend, leading to many typical application cases in the medical field (Long et al., 2015; Ronneberger et al., 2015; Oktay et al., 2018; Chen et al., 2021; Bi et al., 2023). However, most studies have focused on grayscale images, using only single-channel data as network inputs, with fewer studies addressing three-channel data segmentation based on UE images. One existing study (Liu Y. et al., 2022) introduces multiple skeleton networks to evaluate the segmentation performance of U-shaped model structures on the BUE dataset. This study also designs a context extractor at the bottleneck and employs an attention gate (AG) (Oktay et al., 2018) in the skip connections to suppress irrelevant information in the image. The proposed ACE-Net examines the impact of model structure changes on segmentation performance. Unfortunately, this model overlooks the channel features in the middle layer and relies solely on the soft attention mechanism for feature correction. Additionally, the traditional decoder structure is insufficient for fully recovering the features of the elastography image, indicating that the segmentation performance on mediastinal lymph nodes needs further improvement.

On the one hand, traditional ultrasound images suffer from low contrast and high noise, leading to blurred node edges and abnormal boundary changes (Xian et al., 2018; Liu et al., 2019; Chen et al., 2022). On the other hand, UE images with added pseudo color can assist physicians in locating the approximate position of nodules. However, they do not resolve the issues inherent in traditional ultrasound images and introduce additional challenges. Specifically, the pseudo colors obscure the texture information of mediastinal lymph nodes, making it more difficult to capture their actual boundaries, particularly for the accurate segmentation of small mediastinal lymph nodes. Therefore, we combine the attention mechanism and vision transformer (ViT) to conduct an in-depth study of mediastinal lymph node segmentation in bronchial ultrasound elastography images. The main contributions of this research are summarized as follows:

- We design a dual-stream encoder (DSE) combining ConvNext and a lightweight ViT to effectively extract both global and local features from UE images.
- We propose a hybrid attention module (HAM) at the bottleneck to optimize the transmission of high-dimensional features.
- We introduce a feature-enhanced residual decoder (FRD) to recover information and fully fuse the intermediate features of the encoder and decoder using attention and residual structures.
- We use Grad-CAM to visualize heat maps of class activation at different stages of the model, providing insights into the action mechanisms.

## 2 Related work

### 2.1 Medical image segmentation based deep learning

In the early stages of medical image segmentation, traditional methods primarily relied on thresholding, region, edge detection, clustering, and deformable models (Tsai et al., 2003). With the advancement of deep learning, fully convolutional networks (FCNs) (Long et al., 2015) emerged as the most classic segmentation models. FCNs address the limitations of convolutional neural networks (CNNs) in fine-grained image segmentation by replacing fully connected layers with convolutional layers, enabling pixel-level classification to achieve target segmentation. U-Net (Ronneberger et al., 2015) employs a symmetric U-shaped encoder-decoder structure and is widely used in medical image segmentation. Each layer introduces skip connections that combine intermediate features from the encoder and decoder, reducing feature loss and making it particularly suitable for small sample datasets, thereby achieving faster and more efficient segmentation.

There are many variants of U-Net. To enhance the feature extraction capabilities of the model, Dense-UNet (Cai et al., 2020) uses a densely connected network as the decoder, effectively segmenting multiphoton live cell images. To improve the sensitivity to subtle boundaries, Iter-Net (Li et al., 2020) chains U-Net structures together, achieving retinal fundus vessel segmentation by analyzing U-Net structures of different sizes. However, these studies fail to capture contextual features from a global perspective, focusing primarily on spatial domain dependencies.

Recently, researchers have integrated vision transformers (ViT) (Dosovitskiy et al., 2020) into U-Net to enhance feature extraction. For example, Trans-UNet and Swin-UNet have demonstrated impressive performance and accuracy in medical image segmentation. Lin et al. (2023) explored the relationships among CNNs, ViT, and traditional operators, proposing CTO, which performed exceptionally well on multiple medical image segmentation datasets. Bi et al. (2023) combined ViT with deformable convolutions to accurately segment thyroid nodules. These models utilize ViT as an encoder to effectively capture global contextual information while retaining U-Net’s unique multi-scale feature fusion structure. Despite the outstanding performance of ViT, the fixed-size patches limit its ability to perceive fine details and result in high computational costs. Considering the powerful capability of CNNs in capturing local features, we adopt a dual-stream network that combines ViT and CNN to fully exploit the information in medical images.

### 2.2 Attention mechanism

The attention mechanism has shown significant achievements and is widely used in medical image segmentation due to its ability to enhance feature representation and improve the accuracy of segmentation. By selectively focusing on the most relevant parts of the image, attention mechanisms can effectively highlight important regions, such as lesions or tumors, while suppressing irrelevant background noise. For example, Attention U-Net (Oktay et al., 2018) enhances the U-Net by adding AG mechanisms in the skip connections. These AGs re-adjust the encoder’s output features, emphasizing attention weights on the target organ region, thereby improving segmentation accuracy. Lee et al. (2020) proposed an innovative channel attention module that employs a multi-scale averaging pooling operation to cleverly fuse global and local spatial information. MDA-Net (Iqbal and Sharif, 2022) replaces the normal convolution module in U-Net with a multi-scale fusion module and uses a dual attention mechanism to optimize intermediate features in the decoder. Chen et al. (2022) designed a hybrid adaptive attention module for the irregular lesion morphology, which combines channel self-attention and spatial self-attention, and replaced the convolution module in U-Net with it to form AAU-Net. However, given the limitations in feature extraction and enhancement, especially the high-dimensional complex features extracted by DSE, such research may encounter bottlenecks. To address this, we design a hybrid attention module at the bottleneck. This module helps capture more semantically rich features, enables the network to focus on lesion areas, and filters out noise during the feature propagation process.

## 3 Methodology

### 3.1 Overview

**Figure 1:**
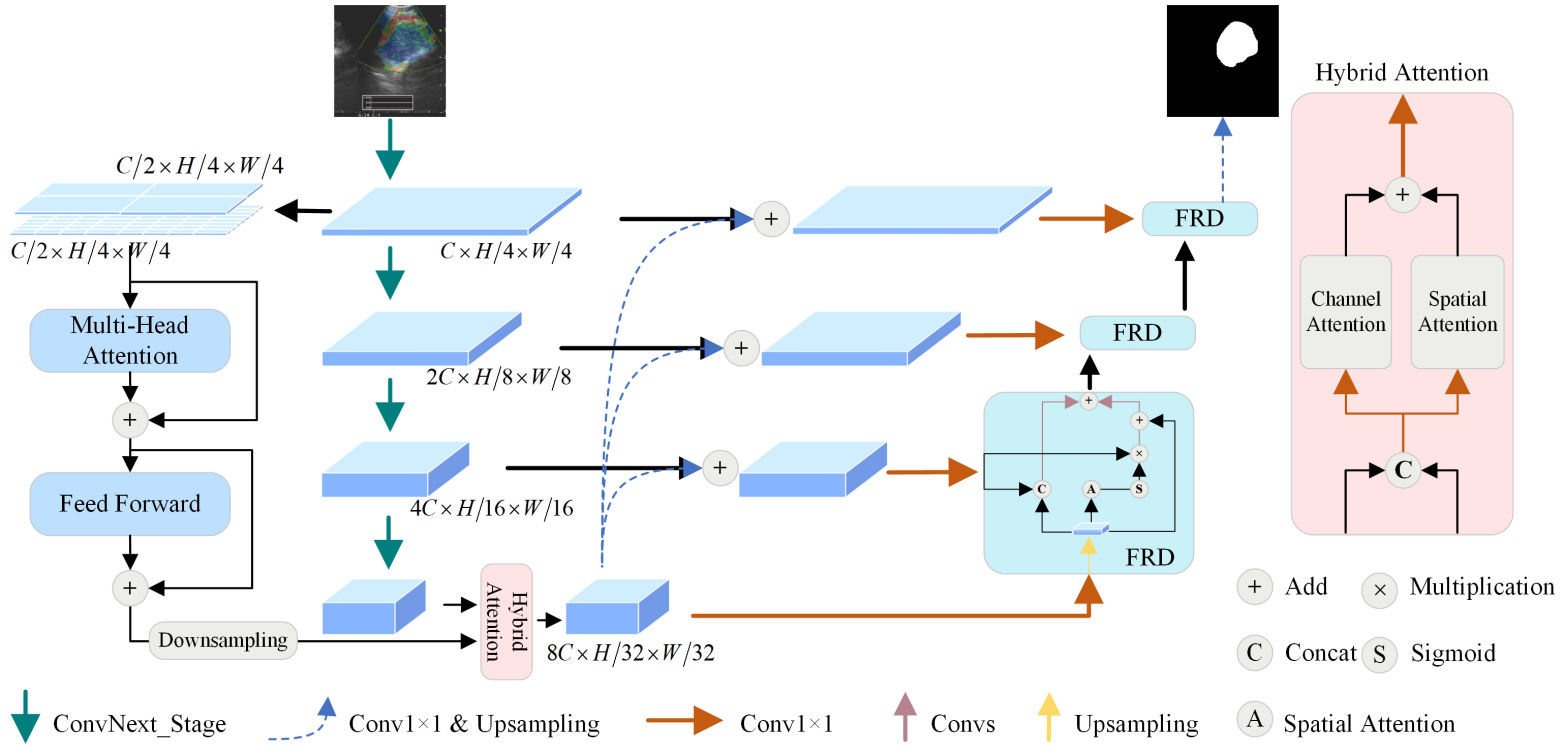
The framework of the proposed dual-stream feature-fusion attention U-Net.

The model proposed mainly contains the following components: dual stream encoder (DSE), hybrid attention module (HAM), and feature-enhanced residual decoder (FRD), and the structure is shown in Figure 1. Firstly, the UE image is fed into the network for multi-order feature extraction using the DSE. Secondly, the features generated by the encoder are optimized using the HAM at the bottleneck. Then, FRD fully fuses the intermediate and underlying features to de-code them. Finally, the features are transformed into a binary map using a convolutional layer and an up-sampling layer. The following section describes in detail the structures in the figure.

### 3.2 Dual-stream encoder

Given that UE images can localize the position of lymph nodes and provide rich channel information, the masking of texture information also leads to the difficulty of performing this task. Therefore, we combine CNNs and ViTs to design a DSE, aiming to effectively capture both local and global features.

A convolutional network encoder is used to capture local feature information of mediastinal lymph nodes from BUE images. Numerous studies (Xie and Richmond, 2018; Raghu et al., 2019) have shown the benefits of pre-trained models, so we use the newly proposed powerful pre-trained ConvNext (Liu Z. et al., 2022) as a convolutional network encoder. It has four outputs are $F_{i},i=1,2,3,4$ , dimensions are C×H/4×W/4 , 2C×H/8×W/8 , 4C×H/16×W/16 and 8C×H/32×W/32 , where C is 128, H and W are both 256.

Vision transformer encoder is used to capture the global feature dependencies of mediastinal lymph nodes to assist the convolutional network encoder for feature extraction. As shown in Figure 1, to minimize model complexity and make full use of intermediate features, $F_{1}$ is used as an input to ViT. Considering the size distribution of the mediastinal lymph node, we used 4×4 and 16×16 patch sizes to divide $F_{1}$ . $F_{1}$ is split equally from the channel dimensions, using dimensionality change and linear layer to divide $F_{1}$ into C/2×H/4P×W/4P,P=4,16 patches, where P denotes the size of the patch. The features are passed into the multi-head attention module, whose main role is to compute the self-attention of the input features to capture the correlation between the features. Specifically, we first use the convolution operation to obtain the query vector Q , the key vector K , and the value vector V of the features. Then the attention score matrix is obtained by the inner product operation between Q and K , which represents the feature-to-feature similarity. Next, the attention score matrix is scaled and probabilization to obtain the attention weight matrix. Finally, the attentional weight matrix is weighted and summed with V to obtain the attentional weighted value matrix. This matrix represents the feature representation obtained after attentional weighting of the input features. Specifically as shown in Equation (1):

(1) $$F_{MHA}=\mathrm{Softmax}\left(\frac{QK^{T}}{\sqrt{d_{k}}}\right)V$$

where $d_{k}$ is the length of K and $F_{MHA}$ is the output of the multi-head attention module.

Send $F_{MHA}$ into the feed forward module to get $F_{FF}$ . The feed forward module consists of two base convolutional modules: a convolutional layer with a kernel of 3 × 3, a batch normalization layer, and a leak ReLU activation function. To further speed up the training, $F_{1}$ , $F_{MHA}$ , and $F_{FF}$ are residually summed to obtain the feature $F_{V}$ extracted by the ViT encoder.

### 3.3 Hybrid attention module

To enhance the extraction of global and local features across various dimensions from the DSE, we design a HAM to optimize the features transmission process by optimizing high-dimensional features at the bottom of the network.

First, by extracting global features using the lightweight ViT, with input and output dimensions unchanged, the resulting $F_{v}$ dimension is C×H/4×W/4 . Then, local features $F_{4}$ are extracted by CNN, with dimensions of 8C×H/32×W/32 . We use down-sampling to resize the $F_{v}$ to the same size as $F_{4}$ . To further enhance the features extracted by the encoder, we concatenate the global feature $F_{v}$ and the local feature $F_{4}$ along the channel dimension and utilize a 1 × 1 convolution to reduce the number of channels to 1/4 of the original, obtaining the feature $F_{f}$ , thereby reducing parameter and computational complexity.

To minimize information loss while enhancing features, we parallelly employ spatial attention modules and channel attention modules to enhance encoder features. The channel attention module first transforms the dimensions of the input feature $F_{f}$ to $C^{\prime }\times H^{\prime }W^{\prime }$ , then generates the attention map $W_{c}$ through matrix multiplication. Finally, $F_{f}$ is multiplied by $W_{c}$ and uses the residual add, resulting in the feature $F_{c}$ enhanced by channel attention, as shown in the formula below:

(2) $$F_{c}=\mathrm{Soft}\max\left(Rs\left(F_{f}\right)•Rs{\left(F_{f}\right)}^{T}\right)\times F_{f}+F_{f}$$

where $Rs\left(•\right)$ denotes the dimensional transformation and $\mathrm{Softmax}\left(•\right)$ denotes the activation function used to normalize the weight values.

For spatial attention, firstly, the channels of $F_{f}$ are reduced to 1 through a 1 × 1 convolution. Then, the Softmax function is applied to normalize the features. Finally, the obtained feature map is multiplied by $F_{f}$ and undergoes residual add, resulting in the feature $F_{s}$ enhanced by spatial attention, as shown in the formula below:

(3) $$F_{s}=\mathrm{Soft}\max\left(\mathrm{Convs}\left(F_{f}\right)\right)\times F_{f}+F_{f}$$

The obtained $F_{c}$ and $F_{s}$ are added and then the channel number is restored using a 1 × 1 convolution, obtaining the enhanced DSE features $F_{cv}$ with dimensions of 8C×H/32×W/32 . This approach comprehensively enhances the image features captured by the feature encoder. Moreover, this parallel attention mechanism reduces the influence of noise, optimizes the feature propagation process at the network bottleneck, and enhances the reliability of the model.

### 3.4 Feature-enhanced residual decoder

To alleviate the situation that ordinary decoder modules may lead to inaccurate segmentation results in the process of feature recovery, we propose the FRD, as shown in Figure 1. Firstly, the feature map $F_{CV}$ is summed with $F_{i},i=1,2,3,4$ to obtain the enhanced fused feature $F_{di},i=1,2,3,4$ by using bilinear interpolation and convolution operations. This preserves the details and location information of the original input image and improves the accuracy of the segmentation results. Then, to reduce the complexity and training difficulty of the model, the number of channels of $F_{di},i=1,2,3,4$ is converted to C/2 using a convolution operation to obtain the feature $F{'}_{di},i=1,2,3,4$ . Finally, $F{'}_{di}$ is passed into the FRD for feature recovery. Anyway, the features of the mediastinal lymph node can be recovered more accurately utilizing FRD, and the accuracy of segmentation results can be improved. The formula is as follows:

(4) $$F{'}_{di}=\mathrm{Con}v_{1\times 1}\left(\mathrm{Up}\left(\mathrm{Con}v_{1\times 1}\left(F_{CV}\right)\right)+F_{i}\right),i=1,2,3,4$$

where $\mathrm{Up}\left(•\right)$ denotes bilinear interpolation for feature transformation and $\mathrm{Con}v_{1\times 1}\left(•\right)$ denotes 1 × 1 convolution for channel conversion.

To make full use of the intermediate features of the model, multiple parallel processing strategies are adopted at the bottom decoding stage. Specifically, there are three branches of processing for $F{'}_{d3}$ and $F{'}_{d4}$ . The first branch performs the bilinear interpolation of $F{'}_{d4}$ with $F{'}_{d3}$ for channel concatenation and passes the result to the convolution module for initial feature recovery. The second branch passes $F{'}_{d4}$ into the spatial attention module to extract the position weight $W_{s}$ , and then performs product operation between $W_{s}$ and $F{'}_{d3}$ to obtain the attention-enhanced features. The third branch residually sums $F{'}_{d4}$ with the features of the first two branches to obtain the output of the decoder module $F_{o3}$ . The formulas for the other decoder modules are shown in Equation (5):

(5) $$F_{oi}=\mathrm{Convs}\left(F{'}_{di}⊕{F^{\mathrm{up}}}_{oi+1}\right)+{F^{\mathrm{up}}}_{oi+1}+SA\left({F^{\mathrm{up}}}_{oi+1}\right)\times F{'}_{di}$$

where $\mathrm{Convs}\left(•\right)$ denotes the base convolution operation; ⊕ denotes channel concatenation; ${F^{\mathrm{up}}}_{oi+1}$ is the output of the decoder after up-sampling; and $SA\left(•\right)$ denotes the spatial attention operation. Through parallel processing and feature fusion, the decoder can fully utilize the features to recover lost details and positional information and improve the accuracy of the segmentation results. This design can effectively compensate for the shortcomings of the common decoder and further optimize the performance of mediastinal lymph node segmentation.

## 4 Experiments

### 4.1 Databases and experimental protocols

#### 4.1.1 Dataset description

A cohort of 206 patients who underwent endobronchial ultrasound-guided trans-bronchial needle aspiration (EBUS-TBNA) was selected from the First Hospital of Nanjing, comprising 141 males and 65 females. We collected 263 UE images of lymph nodes, which were manually delineated by an experienced radiologist. The dataset includes 102 benign and 161 malignant samples. For the experiments, the UE images were uniformly resized to 256 × 256 pixels. The dataset is divided into six equal parts, five of which totalling 219 images are used for training and the other totaling 44 images are used for testing.

We conduct multiple experiments through a six-fold cross-validation approach to fully evaluate the performance of the model. To increase the robustness of the model, we use an online data augmentation method, where the read data are vertically flipped and rotated by a random angle (−30° or 30°) with a probability of 0.5 during the model training iterations.

#### 4.1.2 Implementation details

The proposed DFA-UNet is implemented based on Python 3.7 and Pytorch 1.12. The image processing workstation is equipped with an Intel i9-13900 K CPU and two NVIDIA RTX 4090 GPUs with 24G memory. The initial parameters during model training are obtained by Pytorch default initialization and the Adam optimizer is used to update the network parameters. Specifically, the initial learning rate is set to 0.0001, the weight decay coefficient is 0.1, the learning rate is decayed every 90 rounds of iterations, and the number of iterative training of the model is 190 times in total. Dice (Milletari et al., 2016) is used as the loss function with the following formula:

(6) $$\mathrm{Dice}\;\mathrm{Loss}=1-\frac{2|I_{t}\cap I_{p}|}{\left|I_{t}|+|I_{p}\right|}$$

where $I_{t}$ is the true mask for UE image segmentation and $I_{p}$ is the mask predicted by the model.

#### 4.1.3 Evaluation metrics

To fully demonstrate the segmentation effect of the model, we use the Dice coefficient (Dice), Intersection over Union (IoU), Precision, Specificity, and Hausdorff distance 95^th^ percentile (HD95) (Karimi and Salcudean, 2019) metrics to evaluate DFA-UNet. The Dice is a metric used to measure the similarity of a collection of two samples, in evaluating the performance of image segmentation, Dice can be expressed as:

(7) $$\mathrm{Dice}=\frac{2\times TP}{TP+FP+TP+FN}$$

where *TP*, *FP*, *TN*, and *FN* denote the set of pixel points for true positives, false positives, true negatives, and false negatives. Since the true positives of the background region are not computed during the pixel point classification process, the Dice is suitable for the task of evaluating segmentation targets of varying sizes.

The HD95 is a defined form of the distance between two point sets, calculated as:

(8) $$HD95=\max\left\{d_{tp},d_{\mathrm{pt}}\right\}$$

where $d_{tp}$ denotes the 95% quantile of the farthest distance from $I_{t}$ to $I_{p}$ , and $d_{\mathrm{pt}}$ denotes the 95% quantile of the farthest distance from $I_{p}$ to $I_{t}$ . This metric is more robust to outliers and more suitable for biomedical image segmentation tasks.

In the aforementioned metrics, except for HD95, the value range of the other indicators is [0, 1], with values closer to 1 indicating better model segmentation performance. HD95 has no fixed value range, but lower values of HD95 signify better segmentation performance.

### 4.2 Comparison with the state-of-the-art

#### 4.2.1 Quantitative analysis

To further validate the effectiveness of DFA-UNet on UE images, comparative experiments were conducted with several other models: U-Net (Ronneberger et al., 2015), Att-UNet (Oktay et al., 2018), Seg-Net (Badrinarayanan et al., 2017), DeepLabV3+ (Polat, 2022), Trans-UNet (Chen et al., 2021), U-Net++ (Zhou et al., 2018), BPAT-UNet (Bi et al., 2023), CTO (Lin et al., 2023), and ACE-Net (Liu Y. et al., 2022). The results are presented in Table 1, with the best performance for each metric highlighted in bold.

**Table 1:** Quantitative comparison of our DFA-UNet with other state-of-the-art methods.

Model	Dice (%)	IoU (%)	Pre (%)	HD95	Para (M)	Flops (G)
U-Net	84.61	74.73	84.88	10.39	31.04	54.60
Seg-Net	85.42	75.63	85.54	8.962	29.44	40.01
Att-UNet	85.67	76.04	84.05	9.056	57.16	66.61
U-Net++	85.47	75.91	84.81	9.268	47.18	114.16
Trans-UNet	83.96	73.55	82.25	11.90	105.12	11.89
DeepLabv3+	85.62	76.05	86.07	9.328	**21.54**	45.58
BPAT-UNet	85.90	76.38	84.83	8.725	71.01	64.12
CTO	86.09	76.71	85.05	8.751	60.01	22.59
ACE-Net	86.06	76.55	85.23	8.907	35.01	20.26
DFA-UNet	**86.60**	**77.41**	**86.71**	**8.125**	97.29	**5.27**

Bold values represent the best results.

From Table 1, it can be observed that DFA-UNet outperforms other models in terms of Dice, IoU, Precision, Specificity, and HD95. Specifically, DFA-UNet achieves higher Dice scores compared to U-Net, Seg-Net, Att-UNet, U-Net++, Trans-UNet, DeepLabV3+, BPAT-UNet, CTO, and ACE-Net by 1.99, 1.18, 0.93, 1.13, 2.64, 0.98, 0.70, 0.51, and 0.54%, respectively. Additionally, DFA-UNet shows an improvement of 0.86% in IoU (77.41% vs. 76.55%) and a 1.48% increase in Precision (86.71% vs. 85.23%) compared to ACE-Net. The average improvement in Specificity across the nine compared models is 0.52%. Regarding HD95, DFA-UNet reduces the distance from 10.39 to 8.125 compared to U-Net, with an average reduction of 1.237 across the remaining models, indicating a significant enhancement in segmentation performance. Furthermore, due to the optimization of all parts of U-Net, DFA-UNet, similar to Trans-UNet, BPAT-UNet, CTO, and the other models, achieves better performance compared to U-Net with more parameters. However, it is worth noting that DFA-UNet achieves the best results in model computation within the well-established ConvNext, and also achieves optimal results in segmentation effectiveness.

#### 4.2.2 Qualitative analysis

**Figure 2:**
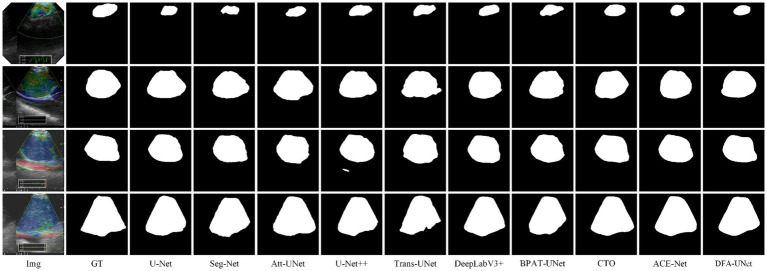
Segmentation results of different models.

To further verify the generality of DFA-UNet for mediastinal lymph node segmentation. We randomly select four segmentation samples of different sizes for qualitative analysis, and their performance is shown in Figure 2.

From Figure 2, it is evident that DFA-UNet exhibits superior segmentation performance for mediastinal lymph nodes of varying sizes. When the target size is small (first row), U-Net, Seg-Net, Att-UNet, BPAT-UNet, CTO-Net, and ACE-Net produce seg-mentation results that are smaller than the actual target, whereas only U-Net++ and DFA-UNet achieve accurate segmentation. For moderately sized targets with relatively simple boundary structures (second row), Trans-UNet, U-Net, Att-UNet, and U-Net++ show significant mis-segmentation, with Trans-UNet performing particularly poorly, as corroborated by the data in Table 1. Additionally, CTO misses part of the segmentation in the lower-right corner of the node. For moderately sized targets with complex boundary structures (third row), Att-UNet, U-Net++, and Trans-UNet fail to accurately segment the lower-right protruding region of the target area, whereas DFA-UNet consistently delivers precise segmentation results. In cases where the target size is large (fourth row), Seg-Net and Trans-UNet exhibit noticeable mis-segmentation in the lower-right depression of the target region, resulting in smaller overall segmentation outputs. U-Net, DeepLabV3+, and BPAT-UNet also show significant mis-segmentation in the low-er-right region. Only CTO-Net, ACE-Net, and DFA-UNet achieve more accurate overall segmentation results, with DFA-UNet providing the best performance across different target sizes and boundary complexities.

#### 4.2.3 Visual analysis

To further explore the underlying mechanisms of DFA-UNet, we employ Grad-CAM (Selvaraju et al., 2017) to visualize the decoding stages of the model. A total of eight models, U-Net, Att-UNet, Seg-Net, Trans-UNet, BPAT-UNet, CTO, ACE-Net, and DFA-UNet, are selected and demonstrated in three stages.

**Figure 3:**
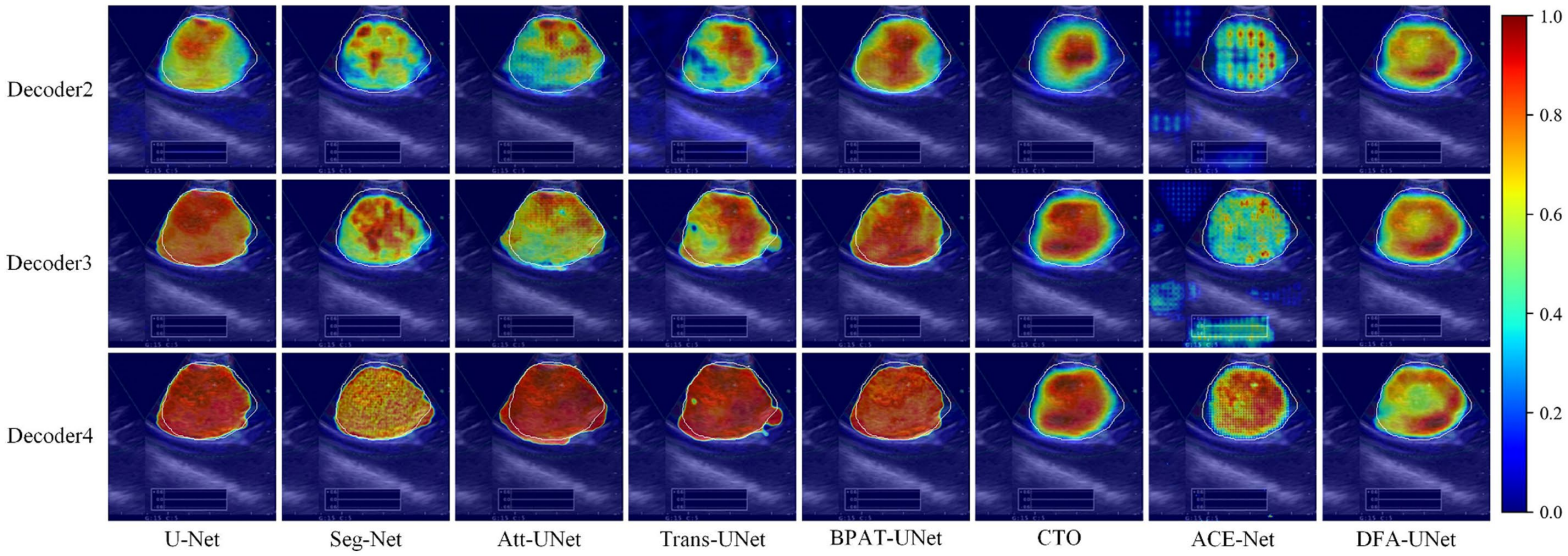
Class activation maps generated by DFA-UNet using Grad-CAM. White contours indicate lymph node locations. Warmer-colored regions correspond to target class labels with higher confidence.

From the overall analysis in Figure 3, it can be seen that the feature extraction capability of the model’s bottom stage determines the feature recovery of the model’s top stage. Specifically, all eight models can roughly locate the real segmentation region in the Decoder2 stage, and further continue to expand outward from the region of interest obtained in the previous stage in the Decoder3 stage. In the Decoder4 stage, the model DFA-UNet shifted the region of interest from the interior to the boundary, which achieved better results in the overall segmentation results. The remaining seven models still further expand the region of interest outwards, resulting in less accurate segmentation results in the higher stages of the model as determined by the target region positioned in the bottom stage of the model.

Secondly, a side-by-side comparison reveals that our DFA-UNet locates the target segmentation region more accurately in the lower stages. During the Decoder2 and Decoder3 phases, the red area representing the region of interest in the DFA-UNet is larger and more uniformly distributed compared to Seg-Net, Att-UNet, Trans-UNet, CTO, and ACE-Net. This uniform distribution closely aligns with the target segmentation region, indicating a better fit.

Finally, the reason for the poor segmentation performance of traditional models can also be analyzed from the figures: either the model’s ability to localize features in the lower layers or its ability to correct feature details in the higher layers is insufficient. Specifically, ACE-Net further extracts high-level semantic information at the bottleneck with the help of a context extractor, which leads to a certain degree of difficulty in re-covering high-level semantic information at the decoder stage, which is manifested in the form of smaller regions of interest in the Decoder2 and Decoder3 stages in Figure 3. Whereas the U-Net model is more accurate in its ability to localize the target segmentation region in the Decoder2 stage, its region of interest is almost unchanged in the Decoder3 and Decoder4 stages, suggesting that the model’s high-level stages are ineffective in correcting feature details. In contrast, DFA-UNet demonstrates superior performance in both the lower and higher stages, resulting in the best overall segmentation outcomes for the region of interest.

#### 4.2.4 Ablation study

We perform ablation studies on each of the key modules of the DFA-UNet. The baseline network is U-Net, which is tested separately with the addition of DSE, HAM, and FRD. As seen in Table 2, the proposed modules promote significant improvements in the baseline network. This fully demonstrates the effectiveness of our DFA-UNet in mediastinal lymph node segmentation.

**Table 2:** Ablation experiment of the proposed DFA-UNet.

DSE-CNN	DSE-ViT	HAM	FRD	Dice (%)	IoU (%)	HD95	Para (M)
				84.61	74.73	10.39	31.04
★				85.07	75.23	9.809	88.58
★	★			85.40	75.63	9.316	89.15
★	★			85.84	76.40	9.014	96.94
★	★	★	★	**86.60**	**77.41**	**8.125**	**97.29**

Bold values represent the best results.

Firstly, using the DSE as the encoder significantly enhances the segmentation performance of the baseline network. The Dice increases by 0.79% (84.61% vs. 85.40%), and the IoU improves by 0.90% (74.73% vs. 75.63%). This notable performance boost is primarily due to the DSE helping the network extract both global and local features. Secondly, incorporating the HAM further improves the feature transfer capability from the DSE, resulting in an additional performance increase. Specifically, the Dice rises from 85.40 to 85.84%, and the HD95 improves from 9.316 to 9.014. Finally, adding the FRD further improves segmentation performance. Compared with the baseline, the Dice is enhanced by 1.99% (84.61% vs. 86.60%), and the HD95 improves by 2.265 (10.39 vs. 8.125). In summary, systematically integrating the feature maps obtained through DSE, HAM, and FRD significantly contributes to the superior performance of our DFA-UNet. Additionally, it is important to note that the parameter count of the lightweight ViT module, DSE-ViT, only occupies a small portion (0.5%) of the total model parameters (88.58 M vs. 97.29 M), confirming its lightweight nature.

#### 4.2.5 Generalization study

To validate the generalization of our DFA-UNet on ultrasound images, we conduct comparative experiments using the BUSI dataset (Al-Dhabyani et al., 2020). This dataset contains 780 breast ultrasound (BUS) images, including 437 benign images, 210 malignant images, and 133 normal images, acquired using the LOGIQ E9 and LOGIQ E9 Agile Ultrasound Systems. Since the primary goal of breast lesion segmentation is to evaluate and identify the distribution of lesions, normal cases without masks were excluded from the BUSI dataset (Ning et al., 2021; Xue et al., 2021). The results of these experiments are presented in Table 3.

**Table 3:** Experiments for generalizability of the proposed DFA-UNet on the BUSI dataset.

Methods	Dice (%)	IoU (%)	Pre (%)	HD95
	70.94	61.77	77.51	30.84
Att-UNet	72.80	63.90	75.49	32.99
DeepLabV3+	78.12	68.75	80.75	21.91
Trans-UNet	76.82	67.41	80.45	21.25
BPAT-UNet	79.37	70.46	81.56	22.66
CTO	78.32	69.61	82.04	20.98
DFA-UNet	**82.68**	**74.59**	**84.44**	**17.01**

Bold values represent the best results.

The results in Table 3 demonstrate that our DFA-UNet achieves state-of-the-art performance in breast ultrasound image segmentation. Specifically, DFA-UNet shows significant improvements over U-Net, with increases of 11.74, 12.82, and 6.93% in Dice, IoU, and Precision, respectively, and a reduction of 13.83 in HD95. When compared with other models, DFA-UNet exhibits an average improvement of 5.59% in Dice, indicating its robust applicability to ultrasound images. Furthermore, comparing the results from Tables 1, 3 reveals that U-Net experiences a 13.67% decrease in Dice when applied to breast ultrasound images, highlighting the increased difficulty of this segmentation task. This also suggests that the color information in ultrasound elastography images aids segmentation. Notably, DFA-UNet shows only a 3.92% decrease in Dice, which underscores its superior generalization capability compared to other models that average a 6.49% decrease. Therefore, DFA-UNet is particularly well-suited for segmenting mediastinal lymph nodes in ultrasound elastography images. This capability has potential clinical value, as it can assist doctors in using ultrasound elastography images for the diagnosis and treatment of lung cancer.

## 5 Conclusion

UE images with rich channel information can provide some guidance for segmentation of the region of interest, but their masking of texture information also leads to the difficulty of performing this task. Additionally, the varying characteristics of different mediastinal lymph node groups further challenge segmentation efforts. To address these issues, we designed a DSE based on ConvNext and a lightweight ViT incorporated into the U-Net. At the bottleneck, we introduced a HAM that combines channel attention with spatial attention to enrich the feature from DSE. The FRD fully fuses intermediate encoder features with decoder output features.

To verify the validity of our DFA-UNet, extensive experiments were conducted to several important conclusions. On the one hand, DFA-UNet employs a dual-stream encoder and an attention enhancement mechanism, which significantly increases the model’s stability. Comparative experiments show that DFA-UNet has clear competitive advantages over current mainstream segmentation models. Class activation maps demonstrate that DFA-UNet achieves superior segmentation sensitivity and completeness by focusing on the content of the region at the lower levels of the network and the boundaries of the region at the higher levels. On the other hand, we optimized various components of the U-Net architecture and presented corresponding ablation experimental results. These findings offer insights for future research aimed at enhancing segmentation performance using U-Net structural variants. This optimization provides a foundation for subsequent studies to explore further improvements in segmentation effectiveness through structural enhancements of U-Net.

In the subsequent research, we will focus on data collection, semi-supervised segmentation tasks, and model optimal structure exploration, to achieve better segmentation results and assist doctors to use UE images for relevant diagnosis and treatment of lung cancer.

## Data availability statement

The original contributions presented in the study are included in the article/supplementary material, further inquiries can be directed to the corresponding authors.

## Author contributions

QZ: Conceptualization, Formal analysis, Investigation, Methodology, Validation, Writing – original draft, Writing – review & editing. YiZ: Conceptualization, Formal analysis, Investigation, Methodology, Validation, Writing – review & editing. NH: Formal analysis, Validation, Writing – review & editing. YaZ: Formal analysis, Investigation, Writing – review & editing. GZ: Funding acquisition, Investigation, Project administration, Supervision, Writing – review & editing. LL: Funding acquisition, Investigation, Project administration, Supervision, Writing – review & editing.
